# Benefit-risk assessment in the context of health technology assessment: concepts, methods and applications

**DOI:** 10.1590/S2237-96222026v35e20251398.en

**Published:** 2026-06-17

**Authors:** Patrícia Coelho de Soarez, Erica Aranha Suzumura, Aline Frossard Ribeiro Bortoluzzi, Ana Carolina Nonato, Fernando Henrique de Albuquerque Maia, Sidney Marcel Domingues, Bruna de Oliveira Ascef, Beate Jahn, Uwe Siebert

**Affiliations:** 1 Universidade de São Paulo, Faculdade de Medicina, São Paulo, SP, Brazil Universidade de São Paulo Faculdade de Medicina São Paulo SP Brazil; 2 UMIT TIROL - University for Health Sciences and Technology, Institute of Public Health, Medical Decision Making and Health Technology Assessment, Hall in Tirol, Tyrol, Austria UMIT TIROL - University for Health Sciences and Technology Institute of Public Health, Medical Decision Making and Health Technology Assessment Hall in Tirol Tyrol Austria; 3 ONCOTYROL - Center for Personalized Cancer Medicine, Division of Health Technology Assessment, Innsbruck, Tyrol, Austria ONCOTYROL - Center for Personalized Cancer Medicine Division of Health Technology Assessment Innsbruck Tyrol Austria; 4 Harvard T.H. Chan School of Public Health, Center for Health Decision Science and Departments of Epidemiology and Health Policy & Management, Boston, MA, United States Harvard T.H. Chan School of Public Health Center for Health Decision Science Departments of Epidemiology and Health Policy & Management Boston MA United States; 5 Harvard Medical School, Mass General Brigham Center for Health Technology Assessment, Boston, MA, United States Harvard Medical School Mass General Brigham Center for Health Technology Assessment Boston MA United States

Most health technologies, such as medications, products, devices and procedures, involve varying levels of risks or harms associated with their use. While the benefits often clearly outweigh the risks, in certain situations the benefit-risk balance can be difficult to determine.

The need for a consistent and systematic approach to determine whether the benefit-risk balance of a single treatment is acceptable, or to verify which treatment has the best benefit-risk balance, has driven the development of a number of methods, ranging from subjective and qualitative judgments to complex quantitative methods. This series of articles in the journal *Revista Epidemiologia e Serviços de Saúde: revista do SUS*, presents methodological approaches for conducting benefit-risk assessments, which also provides the reader with complementary sources of information for more in-depth study on the subject. 

## Benefit-risk assessment concepts

Benefit-risk assessment, also called risk-benefit or benefit-harm assessment, is the process of analyzing and comparing the benefits of a health technology with its potential risks or harms. Its objective is to determine whether the benefits of an intervention outweigh the risks (harms) in a given clinical context, which aids in regulatory, public health and clinical decision-making.

This process is essential for decision-making throughout the entire lifecycle of a health technology, right from development, regulatory approval, post-marketing surveillance, incorporation and reimbursement decisions in health technology assessments, clinical use, through to its becoming obsolete [1-3].

Benefits are the positive or desired effects of the intervention and the expected probability and magnitude of achieving those results for individuals or populations [4], such as disease prevention, mortality reduction, symptom relief and improvement in quality of life.

Risks or harms are the adverse or unwanted effects associated with an intervention, and the likelihood of occurrence or severity of that negative event [4]. They may include experiencing side effects caused by a medication, complications arising from a procedure and psychological harm associated with a diagnosis.

A benefit-risk plan is used to facilitate interpretation of the relationship between benefits and risks. The plan is a quadrant graph that determines whether a technology is favorable or unfavorable compared to another intervention (such as standard treatment or no treatment), considering these two factors (Figure 1).

**Figure 1:**
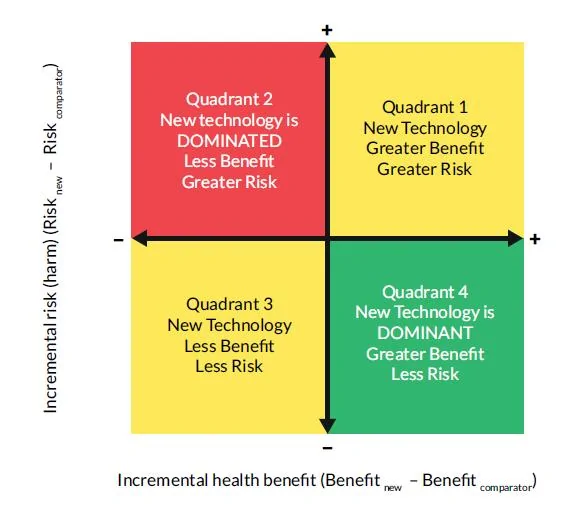
Benefit-Risk Plan.

In Figure 1, quadrant 1 of the graph shows technologies that offer greater benefits but also higher risks, such as some aggressive cancer treatments that can increase survival while potentially causing severe side effects. In these cases, treatment may be justified if the benefits (survival) outweigh the impact of the risks. Conversely, treatment may not be justified if the risks or harms outweigh the benefits, such as experimental treatments and later-line options that show few beneficial results and many side effects.

Quadrant 2 of the graph includes technologies that offer fewer benefits compared to the reference treatment and greater risks, classified as dominated technologies. An example is lumiracoxib, a non-steroidal anti-inflammatory drug indicated for treatment of osteoarthritis, which, when compared to celecoxib, did not demonstrate an analgesic advantage and presented greater hepatotoxicity, leading to its withdrawal from the market in several countries. This is the most unfavorable quadrant.

Quadrant 3 of the graph includes technologies with lower benefits and lower risks compared to the reference treatment. An example is radiotherapy alone for early-stage prostate cancer, which, compared to radical prostatectomy, may be less effective in achieving long-term cancer-free outcomes. However, radiotherapy is associated with lower risks compared to prostatectomy, which involves risks such as incontinence and sexual dysfunction. This trade-off may be acceptable, as radiotherapy offers a less invasive approach with less harm, even if the benefits are slightly lower than those of surgery.

Quadrant 4 of the graph contains dominant technologies, which offer greater benefits and lower risks in relation to the comparator. For example, direct-acting antivirals used in the treatment of hepatitis C, which have higher cure rates and rare adverse events when compared to standard treatment with interferon and ribavirin.

## Methodological approaches to benefit-risk assessment

Methodological approaches for benefit-risk assessment can be classified into: analytical frameworks, metrics, estimation techniques and utility research techniques. Metrics have been defined as measurement systems and classified into two groups: those that establish benefit or risk limits, addressing only one of these dimensions, such as the Number Needed to Treat (NNT) and the Number Needed to Harm (NNH); and those that simultaneously weigh benefits and risks, such as health indices and trade-off indices.

Health indices include standardized and validated measures of quality of life - Quality-Adjusted Life-Year (QALY), Disability-Adjusted Life-Year (DALY). Trade-off indices combine benefits and risks into a single metric - Incremental Net Health Benefit (INHB), Benefit-Risk Ratio (BRR) - which allows for a direct interpretation of whether a therapeutic option is favorable or unfavorable [1,5].

Estimation techniques include general statistical methods, not exclusive to benefit-risk assessment, such as the probabilistic simulation method. Utility research techniques involve elicitation of preferences and values ​​assigned to different outcomes, such as the Discrete Choice Experiment (DCE). Although not specific to benefit-risk assessment, they are used in a complementary way to generate more robust utility estimates and increase transparency in the decision-making process [1,5].

The choice of using analytical frameworks for benefit-risk assessment is justified by their ability to organize and summarize, in a systematic and transparent way, the different dimensions involved in the decision-making process. Analytical frameworks are the central element of any benefit-risk assessment [2]. Unlike isolated metrics, which quantify specific benefits or risks, and estimation or utility elicitation techniques, which play complementary roles in synthesizing evidence and incorporating preferences, analytical frameworks enable integration of multiple clinical outcomes, uncertainties, values and decision-making contexts into an explicit and reproducible process.

Analytical frameworks are subdivided into descriptive or quantitative, and both offer a framework that guides benefit-risk assessment to support decision-making [1], but do not provide mathematical algorithms that automate the decision-making process [6]. The second article in this series addresses descriptive frameworks, while the third article deals with quantitative frameworks. 

Structured approaches to benefit-risk assessment have been used in regulatory decisions and post-marketing surveillance. The first descriptive analytical framework, the Benefit-Risk Action Team (BRAT) framework, was developed in 2005 [7]. The European Medicines Agency has recommended the Problem, Objectives, Alternatives, Consequences, Trade-offs, Uncertainty, Risk and Linked decisions (PrOACT-URL) descriptive analytical framework for new drug applications since 2007 [8]. The Research on Pharmacoepidemiological Outcomes of Therapeutics (PROTECT) initiative has supported the monitoring of benefit-risk assessment of medicines in Europe [9].

In 2009, the United States (US) Food and Drug Administration (FDA) began adopting systematic approaches for benefit-risk assessment in the drug review process and proposed the US Food and Drug Administration - Benefit-Risk Framework (FDA BRF) descriptive analytical framework [10].

In the context of health technology assessment, efforts to use benefit-risk assessment methods are in a preliminary phase compared to the regulatory scenario. Technology effectiveness and safety are assessed, but not compared with each other. A recent scoping review identified only six documents on benefit-risk assessment methods produced by health technology assessment bodies; they do not provide detailed guidance on how to select the best approach or how to conduct benefit-risk assessment in this context [4].

In the regulatory context, benefit-risk analysis is based on the intrinsic efficacy and safety of a health technology, aiming to establish proof of concept and biological viability compared to placebo or the natural history of the disease. Product registration does not require demonstration of superiority over existing alternatives, as its scope is the validation of quality parameters and confirmation that adverse events are acceptable given the observed therapeutic effect.

In health technology assessment, the focus shifts from efficacy to comparative effectiveness and incremental clinical utility. A heath technology must necessarily be compared with another alternative used in the current care pathway in order to measure the real gain in health outcomes. While regulation authorizes the entry of a new technology into the based on its isolated performance, health technology assessment conditions recommendations on the availability of evidence that the new technology offers a superior clinical advantage or, at least, therapeutic equivalence with an optimized safety profile compared to the therapeutic armamentarium established in current care.

As of March 2026, the National Commission for the Incorporation of Technologies in the Brazilian Unified Health System (*Comissão Nacional de Incorporação de Tecnologias no Sistema Único de Saúde*, CONITEC) does not use formal analytical frameworks for the systematic assessment of the benefit-risk balance. Some of its recommendation reports include a section entitled “balance between desirable and undesirable effects”, in which the outcomes associated with the technology assessed are presented in a narrative form, without the explicit use of structured methods for weighting or quantifying these effects.

Figure 2 presents an initial suggestion for selecting methods to perform benefit-risk assessment. It is recommended to use a descriptive analytical framework throughout benefit-risk assessment and to present the results using visual tools, such as a table of effects or a value tree, which will be detailed in the fifth article of this methodological series.

**Figure 2:**
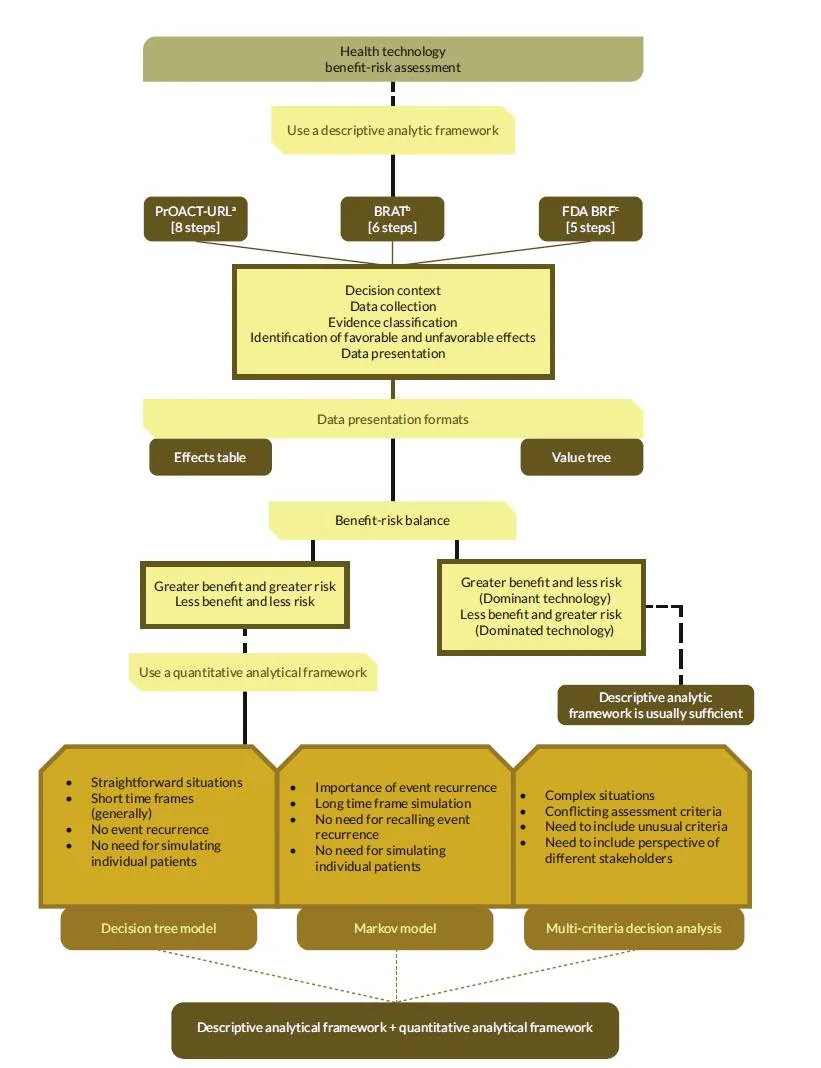
Benefit-risk assessment method selection.

^a^Problem, Objectives, Alternatives, Consequences, Trade-offs, Uncertainty, Risk, and Linked decisions (PrOACT-URL); ^b^Benefit-Risk Action Team (BRAT); ^c^US Food and Drug Administration - Benefit-Risk Framework (FDA BRF).

In the case of technologies falling into Quadrants 2 (dominated technologies) and 4 (dominant technologies), use of a descriptive analytical framework is sufficient for transparently informing the rationale behind the decision. However, in Quadrants 1 and 3, where the benefit-risk balance is not immediately clear and/or stakeholder preferences influence this balance, additional use of quantitative methods may be crucial for decision-making. A trade-off is necessary for these latter quadrants, that is, willingness to accept some harm in exchange for a benefit achieved.

## Why is benefit-risk assessment important in the context of health technology assessment?

Regulatory bodies and health technology assessment agencies have different roles. While regulatory bodies primarily assess the benefit-risk balance of a single treatment, health technology assessment agencies focus primarily on evaluating the comparative effectiveness of a treatment, comparing it with relevant alternatives or with those currently available in the system [11].

Rapid introduction of innovative treatments, such as precision medicine and drugs for rare diseases, has increased the complexity of decision-making in healthcare systems. Safety issues have become more important as regulatory authorities increasingly adopt more flexible standards, processes and evidence requirements for drug approval [11].

Fast-track registration, a process designed to facilitate development and expedite the review and availability of medicines to treat rare conditions and unmet medical needs, reduces the time it takes for new medicines to reach the market. However, faster development and more agile regulatory review are associated with an increase in the number of adverse events and the need for post-marketing safety reviews [12].

Since 2017, when fast-track registration was implemented by the Brazilian National Health Surveillance Agency, the number of applications increased from one in 2018 to 40 in 2022. Many of these registrations were based only on phase II trials or preliminary data from phase III trials to support the decision, which has exposed patients to great uncertainty regarding the efficacy and safety of the medications [13].

Benefit-risk assessment is applied at the time of approval of new technologies and throughout the entire product lifecycle, including post-marketing monitoring. This evolution reflects the need for continuous review, as new evidence emerges and the risk profile of an intervention may change.

In view of the continuous accumulation of information on safety and effectiveness during the post-regulatory approval phase, it is essential to ensure an explicit, transparent and standardized process for continuous data collection and active reassessment of benefits and risks, for management throughout the life cycle of health technologies [11].

Synergy between regulatory and health technology assessment agencies, as well as early dialogue with industry, has been suggested to promote alignment on necessary data that can be shared and used in specific decision-making processes [14,15].

During the decision-making process regarding incorporation of health technologies, it would be important to include a detailed benefit-risk assessment and its results immediately after the synthesis of evidence on the safety and efficacy/effectiveness of the technologies in health technology assessment reports, if possible, using visual tools that facilitate interpretation of the analysis.

We are aware that no single approach is suitable for the multiplicity of populations, diseases, technologies and their clinical applications. Therefore, our intention is not to prescribe or recommend a specific benefit-risk assessment approach, but rather to present the available methods and highlight the importance of their use in future CONITEC reports.
