# Supplementary material for: Benefit-risk assessment in the context of health technology assessment: concepts, methods and applications
Source: Epidemiol Serv Saude. 2026 Jun 17;35:e20251398. doi: 10.1590/S2237-96222026v35e20251398.en (PMC13686407; doi:10.1590/S2237-96222026v35e20251398.en)
Supplement: Translation

# Translation: Avaliação benefício-risco no contexto da avaliação de tecnologias em saúde: conceitos, métodos e aplicações

**DOI:** 10.1590/S2237-96222026v35e20251398.pt

**Authors:** Patrícia Coelho de Soarez, Erica Aranha Suzumura, Aline Frossard Ribeiro Bortoluzzi, Ana Carolina Nonato, Fernando Henrique de Albuquerque Maia, Sidney Marcel Domingues, Bruna de Oliveira Ascef, Beate Jahn, Uwe Siebert

**Affiliations:** 1 Universidade de São Paulo, Faculdade de Medicina, São Paulo, SP, Brasil; 2 UMIT TIROL - University for Health Sciences and Technology, Institute of Public Health, Medical Decision Making and Health Technology Assessment, Hall in Tirol, Tirol, Áustria; 3 ONCOTYROL - Center for Personalized Cancer Medicine, Division of Health Technology Assessment, Innsbruck, Tirol, Áustria; 4 Harvard T.H. Chan School of Public Health, Center for Health Decision Science and Departments of Epidemiology and Health Policy & Management, Boston, MA, Estados Unidos; 5 Harvard Medical School, Mass General Brigham Center for Health Technology Assessment, Boston, MA, Estados Unidos

A maioria das tecnologias em saúde, como medicamentos, produtos, dispositivos e procedimentos, envolve diferentes níveis de riscos ou danos associados ao seu uso. Embora os benefícios frequentemente superem os riscos de forma clara, em determinadas situações o balanço benefício-risco pode ser difícil de ser determinado. 

A necessidade de uma abordagem consistente e sistemática para determinar se o balanço benefício-risco de um único tratamento é aceitável ou para verificar qual tratamento tem o melhor balanço benefício-risco estimulou o desenvolvimento de vários métodos que incluem desde julgamentos subjetivos e qualitativos a métodos quantitativos complexos. Nesta série de artigos na *Revista Epidemiologia e Serviços de Saúde: revista do SUS*, são apresentadas as abordagens metodológicas para realização de avaliações benefício-risco, o que fornece também ao leitor fontes complementares de informação para o aprofundamento no tema.

## Conceitos da avaliação benefício-risco

A avaliação benefício-risco, também chamada de avaliação risco-benefício ou benefício-dano, é o processo de analisar e de comparar os benefícios de uma tecnologia em saúde em relação aos seus riscos ou aos seus danos potenciais. O objetivo dela é determinar se os benefícios de uma intervenção superam os riscos (danos) em determinado contexto clínico, o que auxilia na tomada de decisões regulatórias, de saúde pública e clínicas.

Esse processo é essencial para a tomada de decisão ao longo de todo o ciclo de vida de uma tecnologia em saúde, desde o desenvolvimento, a aprovação regulatória, a vigilância pós-comercialização, as decisões sobre incorporação e reembolso na avaliação de tecnologias em saúde, o uso na prática clínica, até a sua obsolescência [1-3].

Os benefícios são os efeitos positivos ou desejados da intervenção e a probabilidade e a magnitude esperada de atingir esses resultados para indivíduos ou populações [4], como a prevenção de doenças, a redução da mortalidade, o alívio de sintomas e o aumento da qualidade de vida.

Os riscos ou danos são os efeitos adversos ou indesejados associados à intervenção, e a probabilidade de ocorrência ou a severidade desse evento negativo [4]. Eles podem incluir a experiência de efeitos colaterais de um medicamento, as complicações de um procedimento e os danos psicológicos associados a um diagnóstico. 

Para facilitar a interpretação da relação entre os benefícios e os riscos utiliza-se o plano benefício-risco, gráfico de quadrantes que determina se uma tecnologia é favorável ou desfavorável em comparação a outra intervenção (como o tratamento padrão ou nenhum tratamento), com a consideração desses dois fatores (Figura 1). 

**Figura 1:** Plano benefício-risco

No quadrante 1 da Figura 1, estão as tecnologias que oferecem benefícios maiores, mas com riscos mais elevados, como alguns tratamentos oncológicos agressivos que podem aumentar a sobrevida enquanto podem trazer efeitos colaterais severos. Nesses casos, o tratamento pode ser justificado se os benefícios (sobrevida) superarem o impacto dos riscos. Em contrapartida, o tratamento pode não ser justificado se os riscos ou danos superarem os benefícios, como os tratamentos experimentais e opções de última linha que apresentam poucos resultados benéficos e muitos efeitos colaterais. 

No quadrante 2, estão as tecnologias que apresentam benefícios menores em comparação ao tratamento de referência e riscos maiores, classificadas como tecnologias dominadas. Um exemplo é o lumiracoxibe, anti-inflamatório não esteroidal indicado para o tratamento de osteoartrite que, quando comparado ao celecoxibe, não demonstrou vantagem em analgesia e apresentou maior hepatotoxidade, o que levou à sua retirada do mercado em diversos países. Esse é o quadrante mais desfavorável.

No quadrante 3, estão as tecnologias com benefícios menores e riscos menores comparados ao tratamento de referência. Um exemplo é a radioterapia isolada para câncer de próstata em estágio inicial, que, em comparação com a prostatectomia radical, pode ser menos eficaz para alcançar resultados livres de câncer em longo prazo. No entanto, a radioterapia está associada a riscos menores em comparação com a prostatectomia, que envolve riscos como incontinência e disfunção sexual. Essa compensação pode ser aceitável, pois a radioterapia oferece abordagem menos invasiva com menores danos, mesmo que os benefícios sejam ligeiramente inferiores aos da cirurgia.

No quadrante 4, estão as tecnologias dominantes, que oferecem benefícios maiores e riscos menores aos do comparador. Por exemplo, os antivirais de ação direta usados no tratamento de hepatite C, que apresentam taxas de cura maiores e eventos adversos raros quando comparados ao tratamento padrão com interferon e ribavirina.

## Abordagens metodológicas para a avaliação benefício-risco

As abordagens metodológicas para avaliação benefício-risco podem ser classificadas em: estruturas analíticas (*frameworks*), métricas, técnicas de estimativa e técnicas de pesquisa de utilidade. As métricas foram definidas como sistemas de mensuração e classificadas em dois grupos: aquelas que estabelecem limites de benefícios ou de riscos, com abordagem de apenas uma dessas dimensões, como o número necessário para tratar (NNT) e o número necessário para causar dano (NND); e aquelas que ponderam simultaneamente benefícios e riscos, como os índices de saúde e os índices de compensação (*trade-off*). 

Os índices de saúde incluem medidas padronizadas e validadas de qualidade de vida - ano de vida ajustado pela qualidade (*quality-adjusted life-year*, QALY), ano de vida ajustado pela incapacidade (*disability-adjusted life-year*, DALY). Já os índices de compensação (*trade-off*) combinam benefícios e riscos em uma única métrica - benefício líquido em saúde incremental (*incremental net health benefit*, INHB), razão benefício-risco (*benefit risk ratio*, BRR) - o que permite interpretar diretamente se uma opção terapêutica é favorável ou desfavorável [1,5].

As técnicas de estimativa compreendem métodos estatísticos gerais, não exclusivos da avaliação benefício-risco, como o método de simulação probabilística. As técnicas de pesquisa de utilidade envolvem a elicitação de preferências e os valores atribuídos a diferentes desfechos, como o experimento de escolha discreta (*discrete choice experiment*, DCE). Embora não sejam específicas da avaliação benefício-risco, elas são utilizadas de forma complementar para gerar estimativas de utilidade mais robustas e aumentar a transparência no processo de tomada de decisão [1,5].

A escolha por utilizar estruturas analíticas para a avaliação benefício-risco justifica-se por sua capacidade de organizar e de resumir, de forma sistemática e transparente, as diferentes dimensões envolvidas no processo decisório. As estruturas analíticas constituem o elemento central de qualquer avaliação benefício-risco [2]. Diferentemente das métricas isoladas, que quantificam benefícios ou riscos específicos, e das técnicas de estimativa ou de elicitação de utilidades, que desempenham funções complementares na síntese de evidências e na incorporação de preferências, as estruturas analíticas permitem integrar múltiplos desfechos clínicos, incertezas, valores e contextos decisórios em um processo explícito e reprodutível.

As estruturas analíticas são subdivididas em descritivas ou quantitativas e ambas oferecem uma estrutura que orienta a avaliação benefício-risco para apoiar a tomada de decisões [1], mas não fornecem algoritmos matemáticos que automatizam o processo decisório [6]. O segundo artigo desta série aborda as descritivas, e o terceiro artigo trata das quantitativas. 

Abordagens estruturadas para avaliação benefício-risco têm sido utilizadas nas decisões regulatórias e na vigilância pós-comercialização. A primeira estrutura analítica descritiva, a Benefit-Risk Action Team (BRAT), foi desenvolvida em 2005 [7]. A Agência Europeia de Medicamentos tem recomendado a estrutura analítica descritiva Problem, Objectives, Alternatives, Consequences, Trade-offs, Uncertainty, Risk and Linked decisions (PrOACT-URL) para novas aplicações de medicamentos desde 2007 [8]. A iniciativa da Pesquisa Farmacoepidemiológica sobre Resultados de Terapêuticas (PROTECT) tem apoiado o monitoramento da avaliação benefício-risco de medicamentos na Europa [9]. 

Em 2009, a US Food and Drug Administration (FDA), nos Estados Unidos, iniciou a adoção de abordagens sistemáticas para avaliação benefício-risco no processo de revisão de medicamentos e propôs a estrutura analítica descritiva US Food and Drug Administration - Benefit-Risk Framework (FDA BRF) [10]. 

No contexto da avaliação de tecnologias em saúde, os esforços para usar os métodos de avaliação benefício-risco estão em uma fase preliminar em comparação com o cenário regulatório. A efetividade e a segurança das tecnologias são avaliadas, mas não comparadas entre si. Uma revisão de escopo recente identificou apenas seis documentos sobre métodos de avaliação benefício-risco produzidos por órgãos de avaliação de tecnologias em saúde; eles não fornecem orientações detalhadas sobre como selecionar a melhor abordagem e como realizar a avaliação benefício-risco nesse contexto [4].

No âmbito regulatório, a análise benefício-risco pauta-se na eficácia e na segurança intrínsecas da tecnologia em saúde, com vistas a estabelecer a prova de conceito e a viabilidade biológica frente ao placebo ou à história natural da doença. O registro sanitário não exige a demonstração de superioridade em relação às alternativas existentes, pois seu escopo é a validação de parâmetros de qualidade e a confirmação de que os eventos adversos são aceitáveis diante do efeito terapêutico observado. 

Nos órgãos de avaliação de tecnologias em saúde, o foco desloca-se da eficácia para a efetividade comparativa e a utilidade clínica incremental. A tecnologia em saúde deve obrigatoriamente ser confrontada com o comparador utilizado na linha de cuidado vigente, a fim de mensurar o ganho real em desfechos em saúde. Enquanto a regulação autoriza a entrada de uma nova tecnologia baseada em sua performance isolada, a avaliação de tecnologias em saúde condiciona a recomendação à evidência de que a nova tecnologia oferece uma vantagem clínica superior ou, no mínimo, uma equivalência terapêutica com perfil de segurança otimizado em relação ao arsenal terapêutico estabelecido no cuidado vigente.

Até março de 2026, a Comissão Nacional de Incorporação de Tecnologias no Sistema Único de Saúde (Conitec) não utiliza estruturas analíticas formais para a avaliação sistemática do balanço benefício-risco. Em alguns relatórios de recomendação, observa-se a inclusão de uma seção nomeada “balanço entre efeitos desejáveis e indesejáveis”, na qual os desfechos associados à tecnologia avaliada são apresentados de forma narrativa, sem o emprego explícito de métodos estruturados para ponderação ou quantificação desses efeitos.

A Figura 2, apresenta uma sugestão inicial de seleção dos métodos para realizar a avaliação benefício-risco. Recomenda-se utilizar uma estrutura analítica descritiva em toda avaliação benefício-risco e apresentar os resultados por meio de ferramentas visuais, como tabela de efeitos ou árvore de valor, que serão detalhadas no quinto artigo desta série metodológica.

**Figura 2:** Seleção de métodos de avaliação benefício-risco

^a^Problem, Objectives, Alternatives, Consequences, Trade-offs, Uncertainty, Risk, and Linked decisions (PrOACT-URL); ^b^Benefit-Risk Action Team (BRAT); ^c^US Food and Drug Administration - Benefit-Risk Framework (FDA BRF).

Nos casos das tecnologias situadas nos quadrantes 2 (tecnologias dominadas) e 4 (tecnologias dominantes), o uso de uma estrutura analítica descritiva é suficiente para informar de forma transparente o racional da decisão. Porém, nos quadrantes 1 e 3, onde o balanço benefício-risco não é imediatamente claro e/ou as preferências das partes interessadas influenciam esse balanço, o uso adicional de métodos quantitativos pode ser crucial para a tomada de decisão. Nesses quadrantes, uma compensação é necessária, ou seja, a disposição para aceitar algum dano em troca de um benefício alcançado.

## Por que a avaliação benefício-risco é importante no contexto da avaliação de tecnologias em saúde?

Órgãos reguladores e agências de avaliação de tecnologias em saúde possuem diferentes papéis. Enquanto os órgãos reguladores avaliam principalmente o balanço benefício-risco de um único tratamento, as agências de avaliação de tecnologias em saúde concentram-se principalmente em avaliar a efetividade comparativa de um tratamento, comparam-no com alternativas relevantes ou com aquelas disponíveis atualmente no sistema [11].

A introdução rápida de tratamentos inovadores, como os da medicina de precisão e os medicamentos para doenças raras, tem aumentado a complexidade na tomada de decisões nos sistemas de saúde. Questões de segurança tornaram-se mais importantes à medida que as autoridades regulatórias adotam cada vez mais padrões, processos e requisitos de evidência flexíveis para a aprovação de medicamentos [11].

O registro acelerado (*fast-track*), processo criado para facilitar o desenvolvimento e agilizar a revisão e a disponibilidade de medicamentos para tratar condições raras e necessidades médicas não atendidas, reduz o tempo de novos medicamentos chegarem ao mercado. O desenvolvimento mais rápido e a revisão regulatória mais ágil, contudo, estão associados ao aumento no número de eventos adversos e na necessidade de revisões de segurança pós-comercialização [12].

Desde 2017, quando o registro acelerado foi implementado pela Agência Nacional de Vigilância Sanitária no Brasil, o número de solicitações aumentou de uma, em 2018, para 40, em 2022. Muitos desses registros basearam-se apenas em estudos de fase II ou em dados preliminares de estudos de fase III para embasar a decisão, o que expôs os pacientes a grande incerteza quanto à eficácia e à segurança dos medicamentos [13].

A avaliação benefício-risco é aplicada no momento de aprovação de novas tecnologias e ao longo de todo o ciclo de vida do produto, com a inclusão do monitoramento pós-comercialização. Essa evolução reflete a necessidade de revisão contínua, na medida em que novas evidências surgem e o perfil de risco de uma intervenção pode vir a mudar.

Com a contínua acumulação de informações sobre segurança e efetividade durante a fase pós-aprovação regulatória, torna-se essencial para a gestão ao longo do ciclo de vida das tecnologias em saúde um processo explícito, transparente e padronizado para a coleta contínua de dados e a reavaliação ativa dos benefícios e dos riscos [11].

Tem sido sugerida a sinergia entre as agências regulatórias e de avaliação de tecnologias em saúde, bem como o diálogo precoce com a indústria, para promover o alinhamento sobre os dados necessários que podem ser compartilhados e usados nos processos de decisão específicos [14,15].

Durante o processo decisório sobre incorporação de tecnologias em saúde, seria importante a inclusão do detalhamento da avaliação benefício-risco e dos seus resultados logo após a síntese de evidências sobre segurança e eficácia/efetividade das tecnologias nos relatórios de avaliação de tecnologias em saúde, se possível, com o uso de ferramentas visuais que facilitem a interpretação da análise. 

Estamos cientes de que nenhuma abordagem única se adapta à multiplicidade de populações, de doenças, de tecnologias e de suas aplicações clínicas. Portanto, nossa intenção não é prescrever ou recomendar uma abordagem específica de avaliação benefício-risco, mas apresentar os métodos disponíveis e destacar a importância do seu uso nos futuros relatórios da Conitec.
